# African swine fever virus structural protein p17 inhibits IRF3 activation by recruiting host protein PR65A and inducing apoptotic degradation of STING

**DOI:** 10.3389/fmicb.2024.1428233

**Published:** 2024-06-18

**Authors:** Shimin Wang, Zhiyong Xiang, Peng Gao, Yongning Zhang, Lei Zhou, Xinna Ge, Xin Guo, Jun Han, Hanchun Yang

**Affiliations:** ^1^State Key Laboratory of Veterinary Public Health and Safety, College of Veterinary Medicine, China Agricultural University, Beijing, China; ^2^Key Laboratory of Animal Epidemiology of Ministry of Agriculture and Rural Affairs, College of Veterinary Medicine, China Agricultural University, Beijing, China; ^3^College of Veterinary Medicine, Xinjiang Agricultural University, Urumqi, China

**Keywords:** ASFV, p17, IFN-β, PR65A, IFN, apoptosis, STING, MAVS

## Abstract

African swine fever virus (ASFV) is notoriously known for evolving strategies to modulate IFN signaling. Despite lots of efforts, the underlying mechanisms have remained incompletely understood. This study concerns the regulatory role of viral inner membrane protein p17. We found that the ASFV p17 shows a preferential interaction with cGAS-STING-IRF3 pathway, but not the RIG-I-MAVS-NF-κB signaling, and can inhibit both poly(I:C)- and poly(A:T)-induced activation of IRF3, leading to attenuation of IFN-β induction. Mechanistically, p17 interacts with STING and IRF3 and recruits host scaffold protein PR65A, a subunit of cellular phosphatase PP2A, to down-regulate the level of p-IRF3. Also, p17 targets STING for partial degradation via induction of cellular apoptosis that consequently inhibits activation of both p-TBK1 and p-IRF3. Thus, our findings reveal novel regulatory mechanisms for p17 modulation of IFN signaling and shed light on the intricate interplay between ASFV proteins and host immunity.

## Introduction

African swine fever (ASF) is a highly contagious infectious disease affecting domestic pigs and wild boars globally, and currently this acute and hemorrhagic disease poses a significant threat to the world swine industry (Kleiboeker et al., 1998). The etiological agent of this disease is the African swine fever virus (ASFV), a member of the nucleocytoplasmic large DNA virus (NCLDV) group and the sole member of the *Asfarviridae* family within the genus Asfivirus (Chapman et al., 2011). ASFV has a large genomic size of 170–195 kb that is AT-rich and encodes more than 160 viral proteins with more than 50 structural proteins (Wang G. et al., 2021). The huge coding capacity and the complexed virion structure represent a formidable challenge to understand the ASFV biology and pathogenesis. So far, the biological functions of most of the vital proteins await to be dissected (Alejo et al., 2018). In addition, the multifaceted nature of ASFV genetic compositions highlights the challenges associated with combatting the disease, emphasizing the necessity for extensive research.

Modulation of host immunity is a prominent feature of ASFV, and this virus has evolved diverse strategies to counteract the host immunity, particularly concerning the cellular interferon signaling, a most critical line of host cellular defenses against invading organisms (Afe et al., 2023; Niu et al., 2023). Recognition of viral pathogen-associated molecular pattern (PAMP), for example, the viral nucleic acids, by pattern recognition receptors (PRR) such as RIG-I/MDA-5, TLR, and cGAS leads to activation of transcription factors (e.g., IRF3, IRF7, NF-κB, etc.), resulting in production of interferons (Fan et al., 2021). The interferons in turn activate the downstream JAK-STAT signaling via binding to its membrane receptors in a paracrine or autocrine manner, resulting in the production of a diverse array of interferon-stimulated genes (ISG) and thus the establishment of antiviral status (Wang T. Y. et al., 2021). ASFV can impede interferon (IFN) activity via either blocking upstream pathways of IFN production or by attenuating the biological effects of IFNs via targeting downstream pathway, ultimately leading to reduced expression of interferon-stimulated genes (ISGs) (Liu et al., 2021; Dodantenna et al., 2022; Zhang et al., 2022; Li Y. H. et al., 2023). Among these mechanisms, the cGAS-STING signaling stands out as one of the extensively studied pathway, and the past studies have identified more than 30 ASFV proteins targeting this pathway, including pMGF360-11L (Yang et al., 2022b), pMGF505-11R (Yang et al., 2021), pDP96R (Wang et al., 2018), pE248R (Liu et al., 2022), pC129R, pEP364R (Dodantenna et al., 2022), pCD2v (Huang et al., 2023), pE184L (Zhu et al., 2023), pA137R (Sun et al., 2022), pA151R (Li et al., 2024), pI215L (Huang et al., 2021), pI226R (Hong et al., 2022), pM1249L (Cui et al., 2022), pMGF505-7R (Li D. et al., 2021; Li J. et al., 2021; Yang et al., 2022a), pH240R (Ye et al., 2023), pD345L (Chen et al., 2022), pS273R (Luo et al., 2022; Li H. et al., 2023), pMGF360-13L (Luo et al., 2023), and so on. Additionally, ASFV AT-rich DNA genome can serve as a template for RNA polymerase III, generating 5′-ppp-RNA that can be sensed by RIG-I/MDA-5. ASFV-encoded proteins also influence the cytoplasmic RNA-activated IFN pathway (Ran et al., 2022). Thus, there is an intricate and sophisticated interplay between ASFV and its target host.

This study concerns the viral protein p17 that is encoded by D117L and is a crucial inner membrane protein with a size of 117 amino acids. P17 is a type II membrane protein, resides in ER-Golgi membranes, and plays an essential role in virus assembly (Suárez et al., 2010). P17 is a multifunctional protein and is involved in multiple processes, including inhibition of the IFN-β promoter (Zheng et al., 2022), inhibition of protein synthesis (Shen et al., 2021), induction of ER stress (Xia et al., 2020) and mitophagy (Hu et al., 2023). In this study, we unveiled a novel mechanism by which p17 inhibits IFN-β promoter activation. We found that it recruits PR65A, a scaffold protein and a subunit of serine-threonine protein phosphatase 2 A (PP2A), to regulate the phosphorylation status of IRF3. In addition, p17 inhibits p-IRF3 production through STING degradation induced by cell apoptosis. These findings add novel insight into the mechanisms of how p17 downregulates IFN responses.

## Materials and methods

### Cell culture and transfection

The cell lines utilized in this study include HeLa (ATCC CCL-2) and HEK293T (ATCC CRL-3216) that were cultured in DMEM supplemented with 10% FBS. PAMs and WSL cells were maintained in RPMI 1640 medium with 10% FBS (Gibco, NY, United States). All cells were cultured at 37°C in a CO_2_ incubator with 5% CO_2_. Plasmid transfection for PAMs, and WSL cells was carried out using Lipofectamine LTX Reagent (15338100, Thermo Fisher), while for HeLa cells, Lipofectamine 2000 Reagent (11668019, Thermo Fisher) was utilized. Transfections in HEK293T cells employed PEI Transfection Reagent (9002-98-6, Sigma).

### Plasmids and antibodies

A variety of plasmids, including HA-cGAS, HA-STING, HA-TBK1, HA-IRF3, HA-RIG-I-N, HA-MAVS, HA-IRF3/5D, HA-PR65A, p17-myc, and p17-mCherry, were constructed by standard molecular biology by cloning the corresponding gene into the pCAGGS-MCS. Plasmids for IFNβ-Luciferase, PGK-renilla luciferase, PRDIII-Luciferase, and NF-κB-Luciferase were previously described (Brennan et al., 2019; Li A. et al., 2021). The primer sequences used in this study are available in Supplementary Table S1. Antibodies used in this study were obtained from various sources. Rabbit antibodies to cGAS (29958-1-AP), STING (19851-1-AP), IRF3 (11312-1-AP), c-myc (10828-1-AP), and MAVS (14341-1-AP), and mouse-antibodies to TBK1 (67211-1-Ig), STING (66680-1-Ig) and β-actin (66009-1-Ig) were obtained from Proteintech (Wuhan, China). Rabbit antibodies against RIG-I (3743S), MDA5 (5321S), MAVS (24930S), p65 (8242S), TBK1 (38066S) and p-STING (19781S) were obtained from CST (MA, United States). Rabbit monoclonal antibodies to p-TBK1 (AB109272) and p-IRF3 (ab76493) were sourced from Abcam. Rabbit antibodies to PPP2R1A (PR65A) (P30153) were obtained from Solarbio (Beijing, China). Mouse-sourced HA (M180-3) and c-myc (M047-3) monoclonal antibodies were purchased from MBL (TKY, Japan). HRP-labeled goat anti-mouse (ZB-2305) and goat anti-rabbit secondary antibodies (ZB-2301) were acquired from Zsbio (Beijing, China). Alexa Fluor 488/647-labeled goat anti-rabbit (A-11008/A-21245) and Alexa Fluor 568-labeled goat anti-mouses (A-11004) were purchased from Thermo Fisher (United States).

### Luciferase assay

HEK293T cells were co-transfected with HA-cGAS (0.3 μg/mL) and HA-STING (0.3 μg/mL) along with p17-myc plasmids or an empty vector for 24 h. HeLa cells were transfected to express p17-myc for 12 h, followed by transfection with either 2–5 μg/mL of poly(dA:dT) or 10 μg/mL of poly(I:C) for an additional 12–18 h. After transfection, cells were harvested and lysed at room temperature (RT) for 30 min. Post-lysis, the samples underwent centrifugation at 12,000 rpm for 2 min, and the resulting supernatant was transferred to a fresh tube. Luciferase activity was evaluated using the Dual-Luciferase^®^ Reporter Assay kit (E1910, Promega, United States) in accordance with the provided instructions, with normalization of the Firefly Luciferase (Fluc) to Renilla Luciferase (Rluc) ratio against mock controls.

### RNA extraction and RT-qPCR

Total RNA extraction was performed using TRIZOL reagent (15596018CN, Thermo Fisher, United States) following standard procedures, followed by reverse transcription into cDNA using the FastKing cDNA Kit (KR116-01, Tiangen, China). Quantitative PCR (qPCR) with SYBR qPCR Master Mix (Q712, Vazyme, China) was employed to quantify target gene expression, involving a denaturation step at 94°C for 30 s, followed by 40 cycles of 94°C for 5 s and 60°C for 30 s. Relative mRNA levels were normalized to GAPDH or U6 mRNA levels and calculated using the 2^−∆∆CT^ method, with the primer sequences provided in Supplementary Table S1.

### Co-immunoprecipitation analysis

Total cells were harvested and lysed for 30 min using western/IP lysis buffer (P0013, Beyotime, China), supplemented with proteinase inhibitor and phosphorylase inhibitor. After centrifugation at 12,000 rpm for 20 min, the resulting lysate was incubated overnight at 4°C with the specified primary antibody, followed by mixing with SPA/G magnetic beads (88803, Pierce, United States) for 2 h at RT. The beads were washed five times with cold TBST and eluted with Elution buffer (0.1 M glycine, pH 2.0) for 10 min. Then the eluate was collected, neutralized with Neutralization Buffer (1.0 M Tris, pH 7.5), mixed with 1 × SDS loading buffer, followed by incubation in an iron bath for 10 min at 100°C for SDS-PAGE.

### Native-PAGE and western blotting

Cells underwent lysis for 30 min using native lysis buffer (SL1030, Coolaber, China), supplemented with proteinase and phosphorylase inhibitors (Topscience, Shanghai, China). Following centrifugation at 12,000 rpm for 20 min, the supernatants were transferred to new tubes, mixed with 5 × Native-PAGE loading buffer (SL1189, Coolaber, China), and subjected to electrophoresis using Native-PAGE (7.5%). The proteins were subsequently transferred to a PVDF membrane, blocked with 5% BSA in TBST buffer for 2 h at RT, probed overnight at 4°C with the IRF3 primary antibody (11312-1-AP, Proteintech, China), and detected using an HRP-linked secondary antibody. The results were analyzed using an ChemiDoc^™^ MP imaging system (Bio-Rad, ChemiDoc MP, United States).

### Immunofluorescence assay

Transfected cells cultured on coverslips were fixed with 4% paraformaldehyde for 10 min at RT, followed by permeabilization with 0.1% Triton X-100 in PBS for 10 min, and subsequent blocking with 2% BSA in PBS for 30 min. The treated cells were then incubated overnight at 4°C with the specified primary antibody, followed by incubation with the fluorescent secondary antibody for 1 h at RT. Finally, the samples were stained with DAPI. The slides underwent three washes with PBS at each step and were sealed with anti-fade mounting medium (18606, PolySciences, United States).

### RNA interference

siRNAs were designed and synthesized by Genepharma (Nanjing, China), and the sequences are provided in Supplementary Table S1. The specified siRNAs (10 pmol) targeting the gene of interest were transfected into cells for 12 h using Lipofectamine RNAiMAX (13778075, Thermo Fisher), with a scramble siRNA serving as the negative control, followed by the subsequent experiments.

### Flow cytometry

The WSL cells, transfected to express p17-myc for 30 h, underwent trypsinization, harvesting, and cold PBS washing. Subsequently, they were suspended in 100 μL of 1× binding buffer and stained with Annexin-V-FITC and PI solution (A211, Vazyme, China) in a dark environment for 10 min at room temperature (RT). Following staining, 400 μL of 1 × binding buffer was added, and the samples were subjected to analysis using flow cytometry. Mock treatment or exposure to 2 μmol STS for 3 h served as the negative and positive controls, respectively. A total of 10,000 cells were collected for analysis in each sample. The results were analyzed using FlowJo™ software.

### Statistical analysis

Data were presented as mean ± standard deviation (SD). Statistical significance was assessed using student’s *t*-test through GraphPad Prism 9 software (GraphPad, San Diego, CA, United States). A *p*-value of less than 0.05 was considered statistically significant.

## Results

### ASFV p17 suppresses IFN-β promoter activation induced by various stimuli

**Figure 1:**
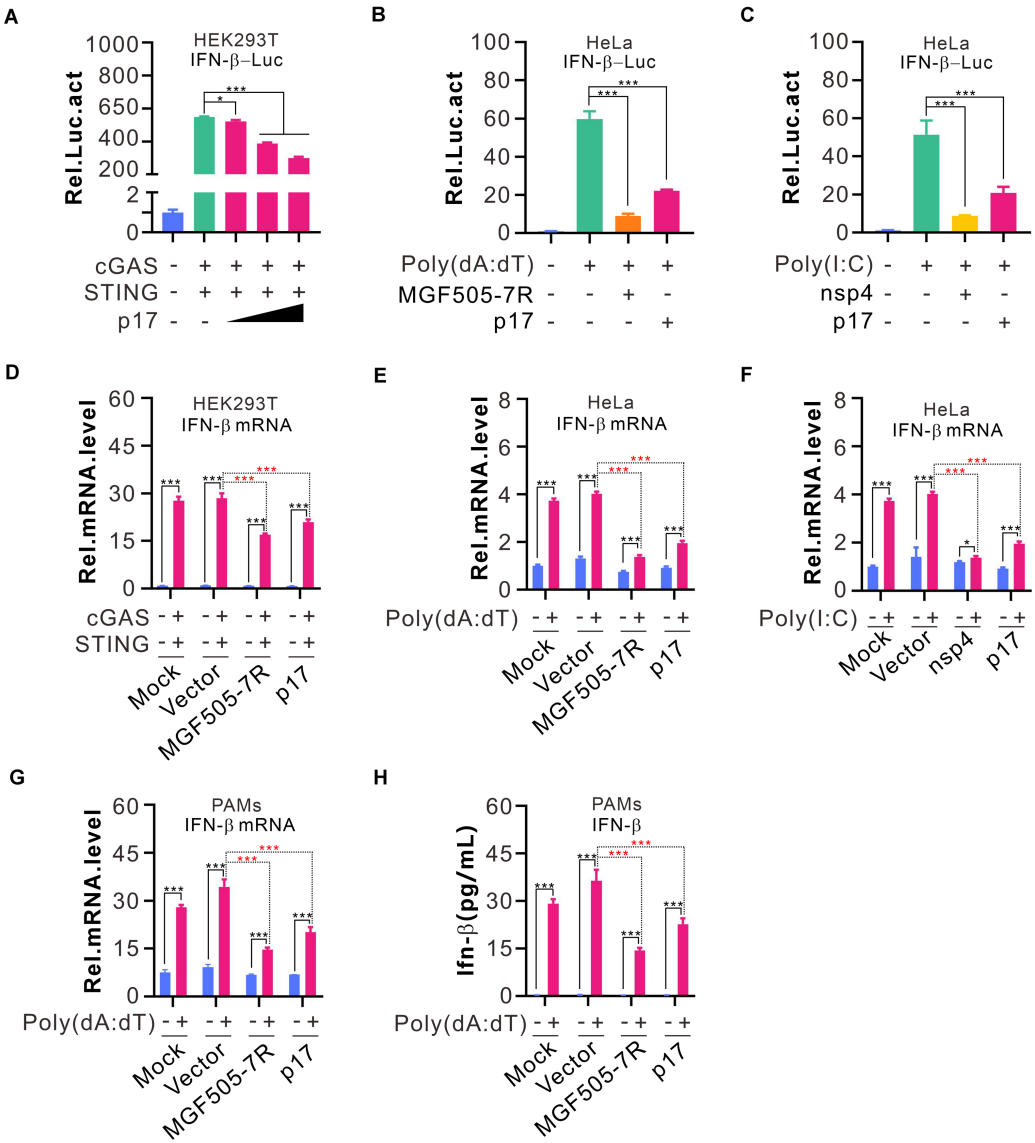
ASFV p17 inhibits IFN-β promoter activation in cells stimulated by various stimuli. **(A–C)** HEK293T cells in 24-well plates were co-transfected to express HA-cGAS (200 ng), HA-STING (200 ng), IFN-β-Luc (300 ng), and pRL-TK (30 ng) in different combinations with or without different amounts of p17-myc for 24 hours. At 18 h post transfection, the HeLa cells were transfected with 2.0 μg poly(dA:dT) or 10.0 μg poly(I:C) for another 12 h before the cells were lysed to assay relative activity of luciferase. **(D–F)** The same as above, except that the total RNA was extracted for quantify the IFN-β mRNA level using qPCR that was normalized against GAPDH. **(G,H)** PAMs treated by 2% DMSO were transfected with indicated plasmids (300 ng) by LTX transfection reagent for 18 h, and then transfected with 4.0 μg poly(dA:dT) for another 12 h. The amount of IFN-β and its mRNA level were assayed by ELISA and qPCR, respectively.

To investigate the effect of p17 on IFN-β promoter activation, we employed the dual luciferase assay (DLR) in which the IFN-β promoter drives the expression of firefly luciferase gene whereas renilla luciferase serves as a transfection control. The HEK293T cells, naturally defective of cGAS-STING, were transfected to coexpress cGAS and STING and various amounts of p17. At 24 h post transfection, the luciferase activity was assayed and normalized against renilla luciferase. Expression alone of cGAS and STING can activate the IFN-β promoter, whereas coexpression of p17 can inhibit this activity in a dose-dependent manner (Figure 1A). P17 also exhibited a similar inhibitory effect on IFN-β promotor activation in HeLa cells stimulated by either poly(dA:dT) or poly(I:C) (Figures 1B,C), in which the ASFV pMGF505-7R and PRRSV nsp4, the two molecules that are known to inhibit IFN-β induction, served as positive controls. We also assessed the expression of IFN-β mRNA by RT-qPCR using GAPDH as house-keeping gene, and the overall trend was the same (Figures 1D–H). Thus, the above results suggest that p17 is a suppressor of IFN-β induction.

### p17 exhibits a differential effect on activation of IRF3 and NF-κB

**Figure 2:**
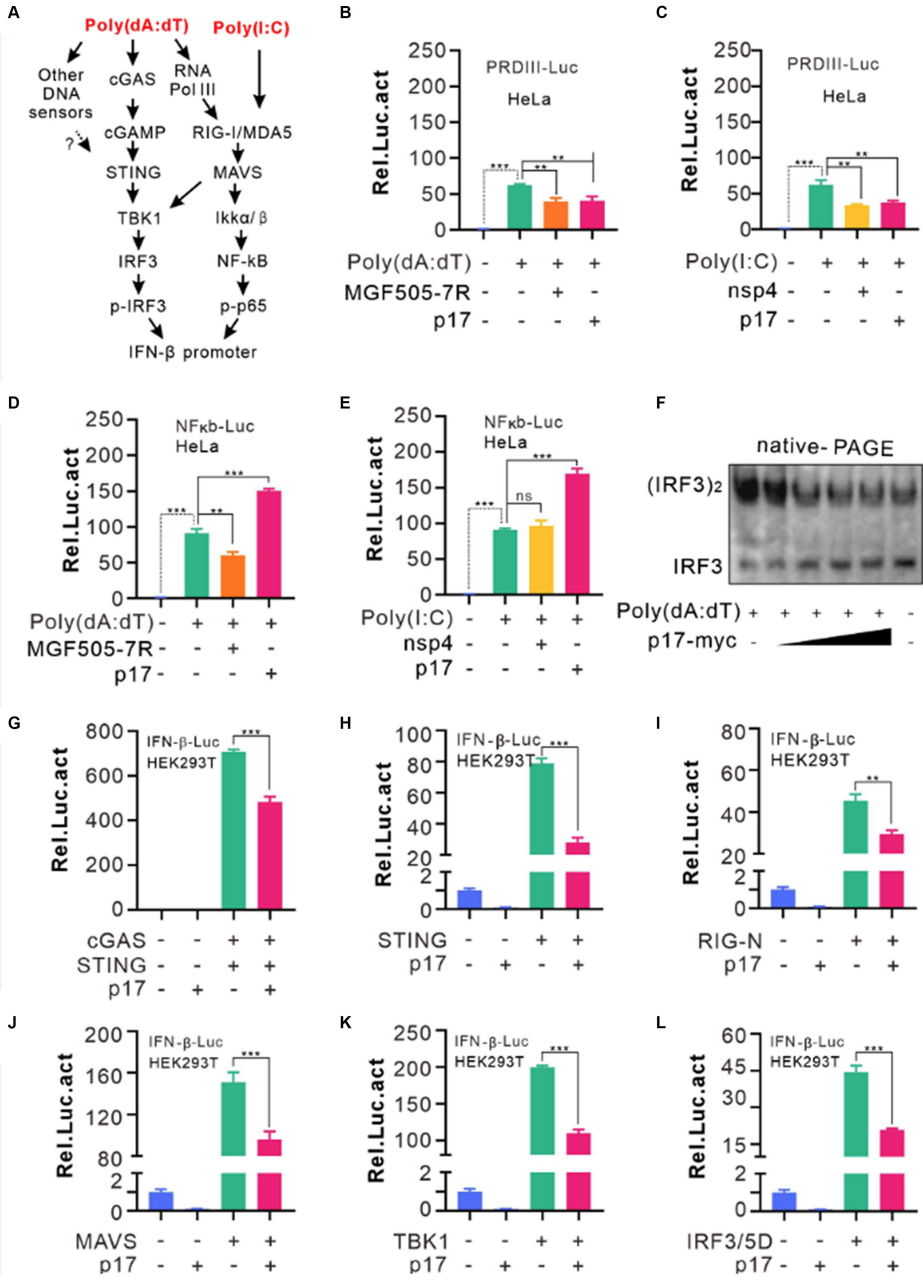
ASFV p17 inhibits IRF3 activation. **(A)** General pathways sensing dsRNAs and dsDNA. **(B–E)** HeLa cells were transfected with the plasmid pPRDIII-luc or pNK-kB-luc together with the plasmid expressing p17, nsp4, for MGF505-7R. At 12 h post transfection, the cells were transfected with poly(dA:dT) or poly(I:C) for 12 h before being lysed for assaying the relative activity of firefly luciferase against renilla luciferase. **(F)** Hela cells transfected with different dosage of p17-myc (0, 0.15, 0.3, 0.6, 1.2 μg) were stimulated by 4 μg poly(dA:dT) for 8 h, and the samples were lysed and resolved by Native-PAGE and blotted with antibodies to IRF3. **(G–L)** HEK293T cells were co-transfected with the plasmids pPGK-renilla-luc and pIFN-b-luc and the plasmid encoding MAVS, TBK1 or IRF3/5D. At 24 h post transfection, the cells were lysed and assayed for luciferase activity. The relative activity was normalized against renilla luciferase.

The fact that p17 inhibited both poly(dA:dT) and poly(I:C)-mediated induction of IFN-β, suggesting that p17 can interfere with the signaling of both MAVS and cGAS-STING pathways. Most often, these cascades culminate in activation of IRF3 or NF-κB or other transcription activators (Figure 2A). Since the IFN-β promotor region often contains the regulatory elements for binding to IRF3 and p65, we used a dual luciferase reporter assay to test the activation status of IRF3 and p65 by constructing pPRD(III-I)-luc (PRDI-III is the binding sequence for IRF3), and pNF-κB-luc plasmids. HeLa cells were first transfected to express either p17-myc or PRRSV nsp4 or ASFV MGF505-7R for 18 h before being stimulated with poly(dA:dT) or poly(I:C) for 12–18 h. We found that the ASFV p17 could inhibit the activation of IRF3 in response to the stimulation by both dsDNA and dsRNA analogs, whereas selectively promoting activation of NF-κB on the other hand (Figures 2B–E). This was further validated by IRF3 dimerization and the nuclear translocation assays, in which IRF3 showed reduced dimerization efficiency in the presence of p17 in a dose-dependent manner (Figure 2F), and p17 could affect the translocation of IRF3 (Supplementary Figure S1). In contrast, p17 did not affect the activation and translocation of NF-κB (Supplementary Figure S2). Overall, our results suggest that poly(A:T) selectively activates IRF3, whereas poly(I:C) can activate both IRF3 and NF-κB, and that p17 exhibits a selective inhibitory effect on IRF3, but not on NF-κB activation.

To further identify the specific signal transduction protein (s) targeted by p17, we used the similar approach. HEK293T cells were transfected to express p17-myc also or an empty vector, or coexpress cGAS-STING, RIG-I-N, STING, MAVS, TBK1, and the active form of IRF3 (IRF3/5D). The results demonstrated that p17 exerted its inhibitory effect in all these cotransfection conditions (Figures 2G–L). Since p17 promoted poly(I:C)-induced NF-κb activation, these combined results suggest that p17 has either multiple targets of cGAS-STING pathway or mainly targets IRF3, but unlikely targets either RIG or MAVS activation.

### ASFV p17 inhibits IRF3 phosphorylation via TBK1-dependent and -independent mechanisms

**Figure 3:**
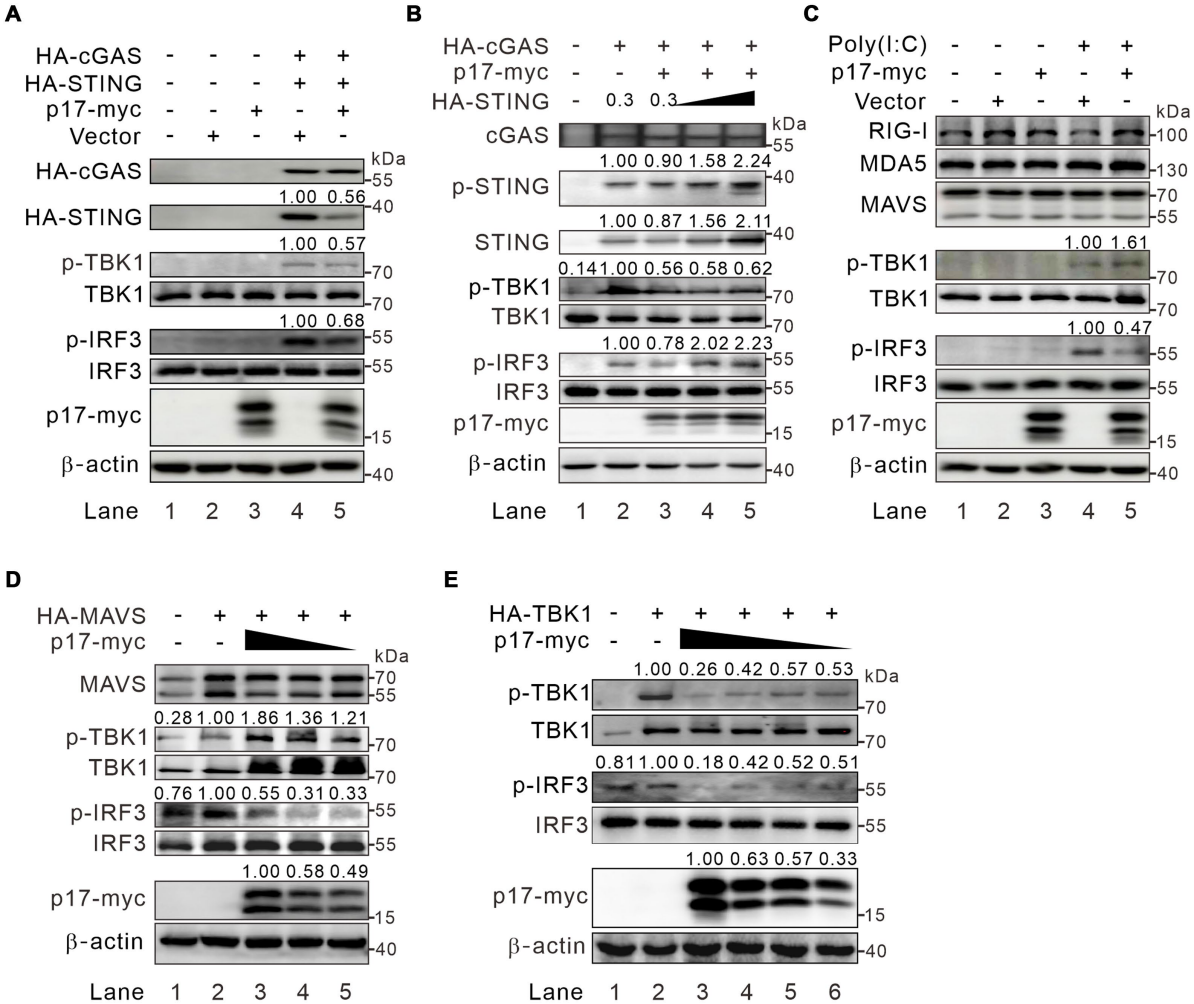
ASFV p17 inhibits IRF3 activation in a p-TBK1-dependent and -independent manner. **(A)** HEK293T cells were transfected to express p17-myc alone or with HA-cGAS (0.3 μg) and HA-STING (0.3 μg). At 24 h post transfection, the cells were lysed and subject to western blot analysis with antibodies to the indicated proteins. **(B)** The same as **(A)**, except that different dose HA-STING (0, 0.3, 0.6, and 1.2 μg) were used. **(C)** HEK293T cells were transfected to express p17-myc. At 24 h post transfection, the cells were transfected with poly(I:C) for 8 h before being subject to western blot analysis with antibodies to the indicated proteins. **(D,E)** HEK293T cells were transfected to express either MAVS or TBK1 together with increasing amount of p17-myc.

We next investigated the phosphorylation status of the signaling molecules in the co-expression assay. The HEK293T cells were cotransfected with p17-myc and either HA-cGAS and HA-STING, or HA-MAVS. The western blot analysis showed that p17 exhibited an inhibitory effect on the phosphorylation of both TBK1 and IRF3 by a reduction of 30–40%, with a concurrent reduction of STING abundance (Figure 3A, lane 5). Overexpression of STING restored p-IRF3 levels, but only partially recovered p-TBK1 levels, amounting to approximately 60% of the total (Figure 3B), suggesting the existence of an additional mechanism, yet to be identified, through which p17 regulates the phosphorylation of TBK1. Additionally, in the RLR-MAVS pathway, although p17 suppressed IRF3 phosphorylation, it paradoxically increased p-TBK1 levels in HEK293T cells overexpressing MAVS (Figure 3D, lanes 3–5). This paradoxical enhancement was further confirmed through poly(I:C) stimulation (Figure 3C, lane 5). Notably, this phenomenon was not observed in cells overexpressing HA-TBK1 (Figure 3E, lanes 2–6), suggesting that the enhancement of p-TBK1 is dependent on MAVS. Consequently, these findings suggest that p17 can inhibit IRF3 activation through mechanisms that are both dependent and independent of p-TBK1 (see the discussion for the explanation).

### ASFV p17 recruits PR65A to regulate the level of p-IRF3 and p-TBK in MAVS-IRF3 pathway

**Figure 4:**
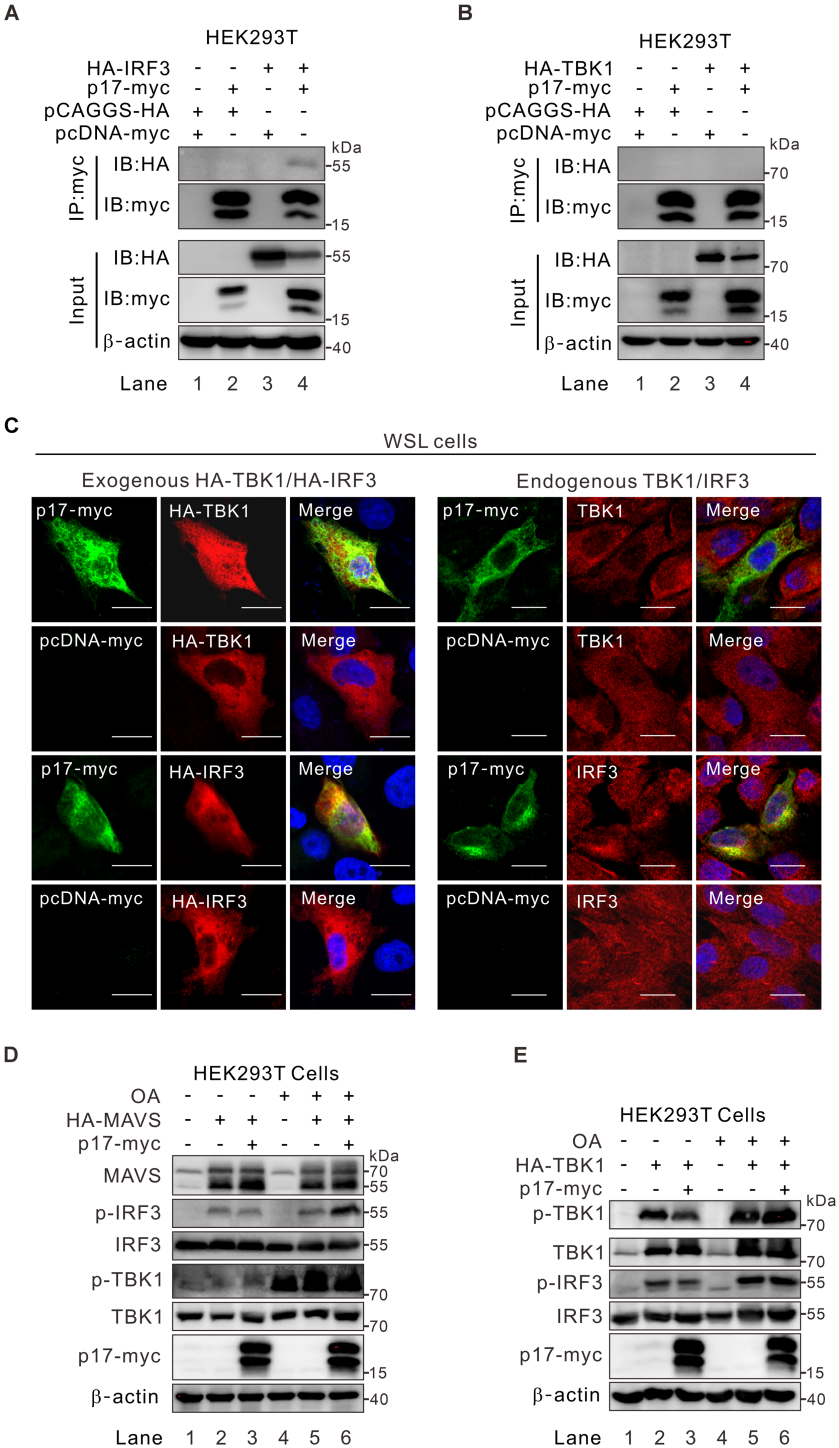
ASFV p17-mediated effect on p-TBK1 and p-IRF3 is regulated by phosphatase activity. **(A,B)** HEK293T cells were co-transfected with plasmid for p17-myc and plasmid for either HA-TBK1, HA-IRF3 or empty vector. At 24 h post transfection, the cells were lysed and analyzed by coimmunoprecipitation with antibodies to myc epitope, followed by western blot analysis with antibodies to the indicated proteins. **(C)** Colocalization analysis of p17 with exogenous and endogenous IRF3 and TBK1 in transfected WSL cells. **(D,E)** HEK293T cells were transfected to express HA-MAVS or HA-TBK1 individually or together with p17-myc, and then treated with OA for 3 h before being assayed by western blot with antibodies to indicated proteins.

Under physiological conditions within cells, the intracellular presence of p-TBK1 is predominantly maintained at a low level, regulated either by dephosphorylation through cellular phosphatases or by degradation of p-TBK1 (McCoy et al., 2008; Cui et al., 2012; Zhao, 2013). Upon cellular stimulation, there was an increase in p-TBK1 levels, which, to some extent, influences the level of p-IRF3. To elucidate the mechanism by which p17 orchestrates the regulation of TBK1 and IRF3 phosphorylation, we tested whether p17 interacts and TBK1 or IRF3 by using IFA and Co-IP assays. The results revealed that the myc antibody to p17-myc could pulldown IRF3 (Figure 4A) and that p17 could partially colocalize with this molecule (Figure 4C), suggesting there is an interaction between these two molecules. Interestingly, p17 did not colocalize (Figure 4C), or interact with TBK1 (Figure 4B, lane 4), suggesting that effect of p17 on TBK1 phosphorylation status is likely indirect. If so, we postulated that p17 may recruit specific phosphatases or related proteins to affect the phosphorylation status of TBK1. To test this hypothesis, HA-MAVS or HA-TBK1 plasmids were co-transfected with p17-myc plasmids into HEK293T cells, followed by treatment for 3 h with 50 nM okadaic acid (OA), an inhibitor of serine/threonine-protein phosphatases. Interestingly, we found that OA treatment was able to inhibit the dephosphorylation of both p-TBK1 and p-IRF3 even in the presence of p17 (Figures 4D,E). Thus, it seems that p17 exerts a certain effect on a phosphatase to downregulate the level of p-TBK1 and p-IRF3.

**Figure 5:**
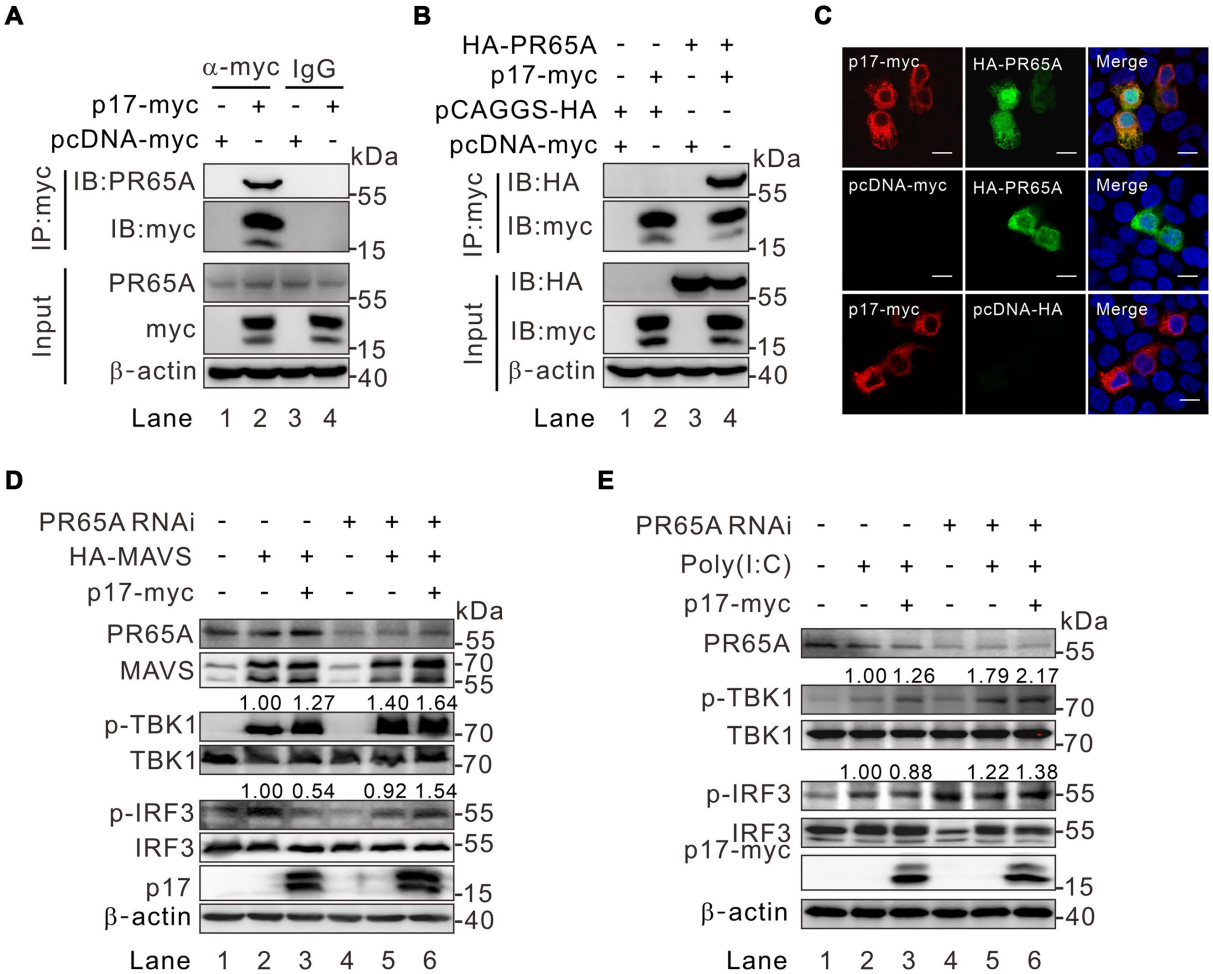
Identification of PR65A as a critical binding partner of p17 for regulating the level of p-IRF3 and p-TBK in the MAVS-IRF3 pathway. **(A,B)** Analysis of the interaction of p17 with endogenous and exogenous PR65A in transfected HEK293T by Co-IP assay. **(C)** Colocalization analysis of p17 with PR65A in transfected HEK293T cells by immunofluorescence assay. **(D)** HEK293T cells were transfected with PR65A siRNA (10 pmol) for 12 h, and then transfected with plasmid for HA-MAVS alone or with plasmid for p17-myc for 24 h. The phosphorylation of TBK1 and IRF3 was detected by western blot analysis. **(E)** The same as **(D)**, except that, at 12 h post transfection, the p17 expressing cells were stimulated with 10 μg poly(I:C) for another 12 h.

To identify the specific phosphatase involved, we overexpressed p17-myc in transfected WSL cells, followed by immunoprecipitation with antibodies to the myc epitope and mass spectrometry analysis. Of 162 host proteins identified, only one protein was associated with the function of protein phosphatase. That is the serine/threonine protein phosphatase 2A 65 kDa regulatory subunit A alpha isoform (PPP2R1A) that is also recognized as PR65A (Supplementary Table S2). Notably, PR65A is a scaffold subunit of protein phosphatase 2A (PP2A). The dimeric complex formed by PR65A and the catalytic subunit can associate with various regulatory subunits, facilitating the dephosphorylation of diverse substrates implicated in cell division and immune-related signaling pathways (Mayer-Jaekel et al., 1993; Wera et al., 1995; Eitelhuber et al., 2011). We further tested the interaction of p17 with this host protein by co-immunoprecipitation (Co-IP) assay and found that antibody to p17-myc could pulldown either the endogenous or the exogenously expressed PR65A (Figures 5A,B). The result from the immunofluorescence assay (IFA) showed that there is a partial colocalization in the cytoplasm between the two proteins (Figure 5C). We further tested the role of PR65A in regulation of TBK1 and IRF3 phosphorylation status by RNAi silencing (Figures 5D,E). Consistent with above results, when PR65A was silenced, it could restore the level of p-IRF3 and further increase the level of p-TBK1 (Figure 5D, lane 6). The results were similar in poly(I:C) treated cells (Figure 5E, lane 6). Thus, the cellular protein PR65A plays an important role in p17-mediated dephosphorylation of IRF3.

### ASFV interacts with STING and inhibits the phosphorylation of TBK1 by recruitment of PR65A in the STING-IRF3 axis

**Figure 6:**
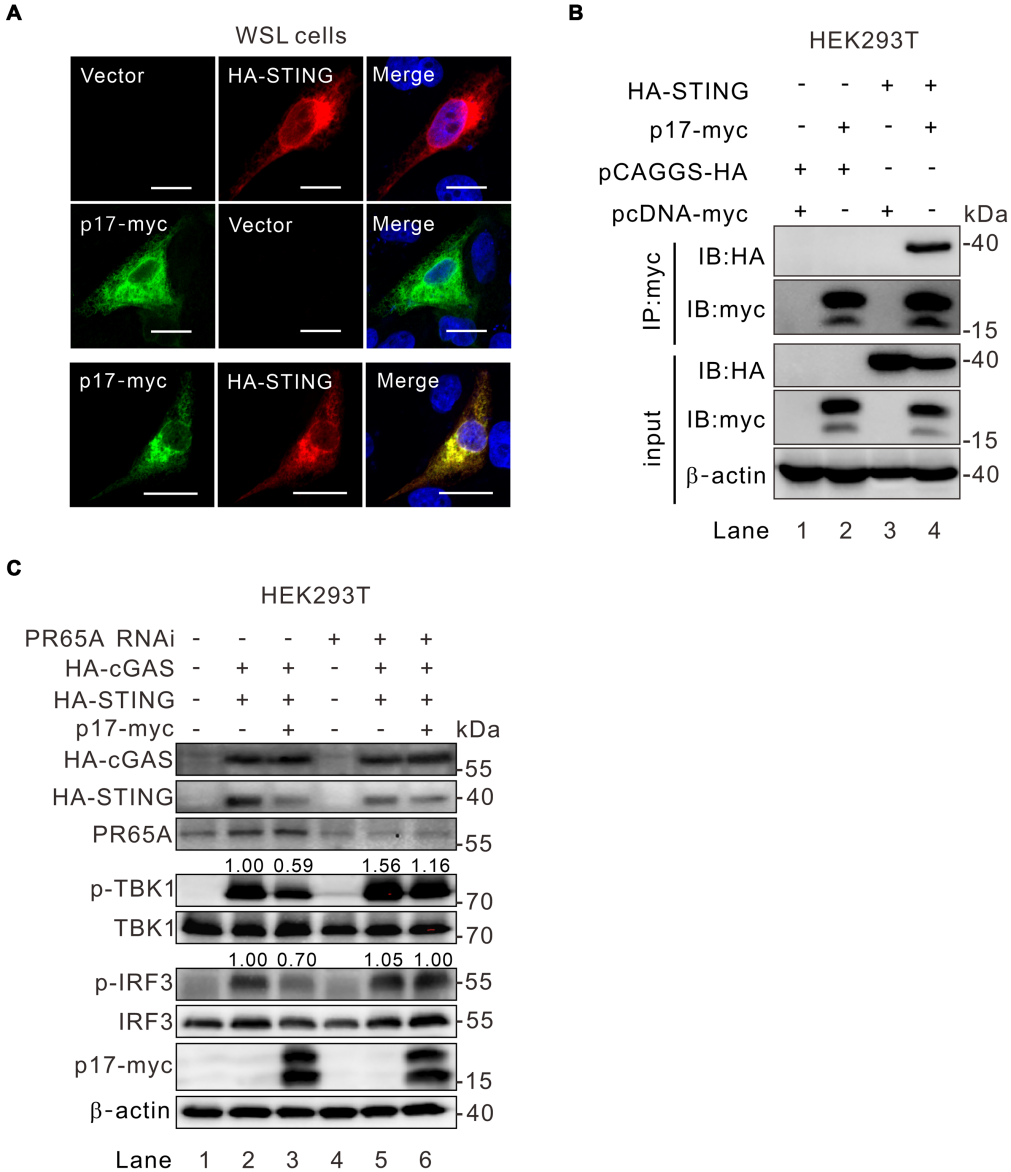
PR65A is critical to ASFV p17 modulation of TBK1 phosphorylation in the cGAS-STING-IRF3 pathway. **(A)** Colocalization analysis of p17 with STING by immunofluorescence assay in transfected WSL cells. **(B)** Interaction analysis of p17 with STING in cotransfected HEK293T cells by Co-IP assay. **(C)** HEK293T cells were transfected with siRNA against PR65A for 12 h, and then transfected to express HA-cGAS and HA-STING along with p17-myc for 24 h, followed by detection of TBK1 and IRF3 phosphorylation by western blot.

The above results (Figure 3B) showed that expression of p17 consistently led to lower levels of p-TBK1 in STING-overexpressing cells, but with an increased level of p-TBK in MAVS overexpressed cells. This discrepancy suggests an unidentified mechanism by which p17 differentially modulates TBK1 phosphorylation. Unlike the mitochondria-resident MAVS, the ER-resident STING was observed to colocalize well with p17 (Figure 6A). In addition, the Co-IP assay showed that there was interaction between p17 and STING (Figure 6B, lane 4). We next tested the role of PR65A in this process by RNAi silencing. Indeed, the inhibition of p-TBK1 and p-IRF3 by p17 within the cGAS-STING pathway was alleviated due to the PR65A knockdown (Figure 6C, lane 6), underscoring the pivotal role of PR65A in p17-mediated down-regulation of p-IRF3 and p-TBK levels. These findings strongly support the idea that ASFV p17 inhibits the phosphorylation of STING-bound TBK1 through PP2A (see more in discussion section).

### ASFV p17 targets STING for degradation via induction of cellular apoptosis

**Figure 7:**
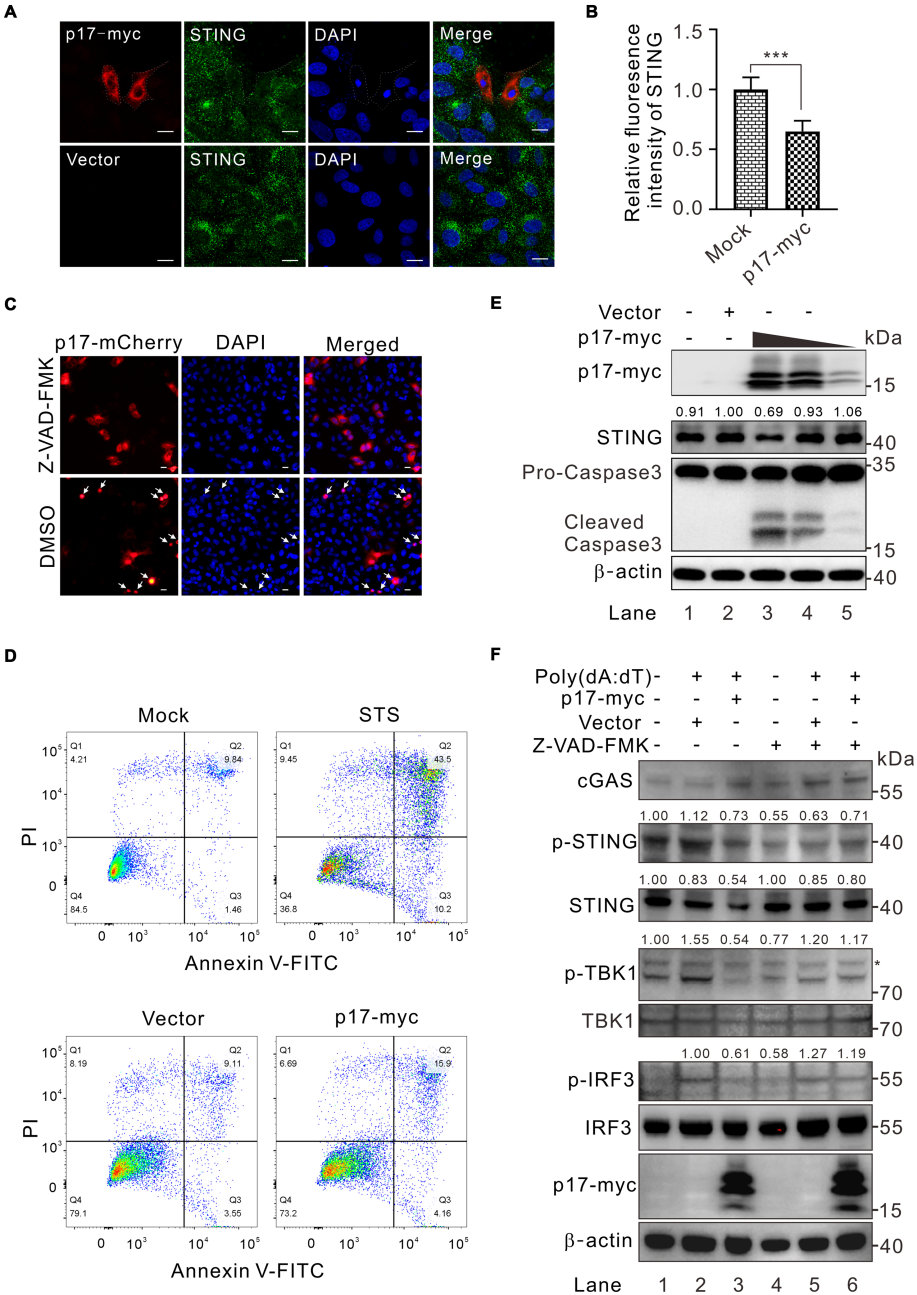
ASFV p17 targets STING for degradation by inducing cellular apoptosis. **(A,B)** Analysis of the effect of p17-myc on the expression of endogenous STING in transfected WSL cells. **(C)** The same as **(A)**, except that the cells were pretreated with a pan-apoptosis inhibitor Z-VAD-FMK and transfected with p17-mCherry plasmid. **(D)** Effect of overexpression of p17 on transfected WSL cells by FACS analysis at 30 h posttransfection. STS treatment served as a positive control. **(E)** Dose-dependent effect of p17 expression on activation of cellular apoptosis and degradation of endogenous STING in transfected WSL cells by western blot. **(F)** Effect of Z-VAD-FMK treatment on the phosphorylation of TBK1 and IRF3 in transfected WSL cells expressing p17-myc, followed by poly(A:T) stimulation for 12 h.

Along the assays, we also observed a reduction of STING abundance in p17 overexpressed cells by western blot analysis (Figures 3A,B), raising the possibility that p17 may also mediate the degradation of STING. We observed that there was a dramatic reduction of endogenous STING fluorescence intensity with a reduction of 40–50% in cells expressing p17 (Figures 7A,B), while qPCR results indicated that p17 had minimal effect on the STING transcription and mRNA nuclear export (Supplementary Figure S3). In particular, this was accompanied by nuclei condensation, indicating the occurrence of cellular apoptosis (Figures 7A–C). Indeed, we found that p17 is an apoptosis inducer, and overexpression of p17 can lead to increased apoptosis, as compared to the mock-treated control (Figure 7D), in which staurosporine (STS) served as a positive control. When an apoptosis inhibitor Z-VAD-FMK was added, it blocks the abnormality of nuclei induced by p17 (Figure 7C). To further validate the claim that p17 induces STING degradation, endogenous STING was examined in WSL cells transfected to express increasing amount of p17. Western blot analysis revealed a dose-dependent degradation of STING by p17 and activation of caspase 3 (Figure 7E, lane 3–5). We also observed the similar effect of p17-mediated degradation on exogenous STING (Supplementary Figure S4). However, when the cells were pretreated with the pan-caspase inhibitor Z-VAD-FMK, it restored not only the STING level in p17-expressing cells, but also partially the level of p-TBK1 and p-IRF3 in cells stimulated with poly(dA:dT) (Figure 7F). Thus, these results indicated that p17 can induce degradation of STING via cellular apoptosis.

## Discussion

ASFV has developed diverse strategies to manipulate host innate immunity, thereby facilitating viral replication (Afe et al., 2023; Niu et al., 2023). Interferon-beta (IFN-β), a potent defense mechanism against viral infections, is typically antagonized or disarmed by the virus upon infection. In this report, we show that ASFV p17 modulates both the RLR-MAVS and cGAS-STING pathways via downregulating the phosphorylation of IRF3 through recruiting local phosphatase PP2A and promoting STING degradation, leading to inhibition of IFN-β induction. The relevant significance/implications are discussed below.

One interesting finding in this study is that p17 exhibited a differential effect over the activation of IRF3 and NF-κB. We found that poly(I:C) could activate both IRF3 and NF-κB pathways, while poly(A:T) mainly activates IRF3 with a very weak action on NF-κB (Supplementary Figures S1, S2). This result is consistent with previous findings that MAVS can recruit both TBK1 and IKK, leading to subsequent phosphorylation of IRF3 and NF-κB, whereas STING primarily recruits TBK1 to activate downstream IRF3 and to a lesser extent NF-κB, in comparison to MAVS-mediated signaling (Liu et al., 2015; de Oliveira Mann et al., 2019; Hopfner and Hornung, 2020; Lv et al., 2023). In this investigation, we observed that p17 selectively inhibits IRF3 activation, but not interferes with the NF-κB activation induced by DNA and RNA analogs (Figures 2B–E). This result is in line with the result that the ER-resident protein p17 mainly interacted with the proteins in the cGAS-STING pathway, such as STING and IRF3, but not with TBK1. The interaction of p17 with these two molecules was evidenced by co-IP and co-localization assays in the transfected cells (Figures 4, 6).

The second discovery is that p17 showed an intriguingly bidirectional regulation of TBK1-phosphorylation. That is, overexpression of MAVS increased the level of p-TBK1 despite the presence of p17, but overexpression of cGAS-STING did not have this effect in the presence of p17 (Figures 3A–D). This paradoxical effect can be explained by that the MAVS-mediated activation of TBK1 mainly takes place on mitochondria, making it physically less accessible to p17, an ER-resident membrane protein. On the other hand, since p17 interacts with STING, when cGAS-STING were over expressed, both TBK1 and IRF3 are recruited to the ER-Golgi complex, make it physically accessible to p17, leading to inhibition of TBK1 activation.

Phosphatases play pivotal roles in maintaining immune homeostasis within host cells and are integral to regulating IFN-β production through diverse mechanisms (Xu et al., 2008; Gabhann et al., 2010; Gu et al., 2014; Sun et al., 2017; Xu et al., 2019). Reports on PP2A, a phosphatase, underscore its significant role in balancing innate immunity by modulating the phosphorylation status of TBK1 and IRF3 (Long et al., 2014; Xu et al., 2019; Zhou et al., 2019). Also, caspase inhibitors can reactivate this signaling in a STING-dependent manner (Tabtieng et al., 2018, 2022). We provide evidence that the downregulation of p-TBK1 and p-IRF3 was executed by at least two possible mechanisms. The first is by recruitment of the cellular factor PR65A, one of the three subunits of cellular phosphatase PP2A. When the expression of PR65A was repressed by RNAi silencing, it reversed the inhibitory effect of p17 on both p-TBK1 and p-IRF3 in the cotransfection assays (Figures 5, 6). The second is to target STING for degradation via induction of cellular apoptosis (Figure 7). We showed that overexpression can lead to cell apoptosis and activation of caspase 3, whereas treatment of the cells with a pan-caspase inhibitor reversed this effect, preventing the degradation of STING even in the presence of p17 (Figure 7).

In addition, in all cases, we found that the presence of p17 could reduce the level of IRF3 phosphorylation. This likely takes place at several manners. P17 can interact with IRF3 and reduce its phosphorylation by recruiting cell phosphatase PP2A (Figure 4). This partially explains why overexpression of MAVS led to an increased level of p-TBK1, but not p-IRF3, as the latter can be recruited away from mitochondria by p17 via an interaction. This also partially explains why simple overexpression of TBK1 did not lead to an increase IRF3 phosphorylation when co-expressed with p17 (Figure 3E).

**Figure 8:**
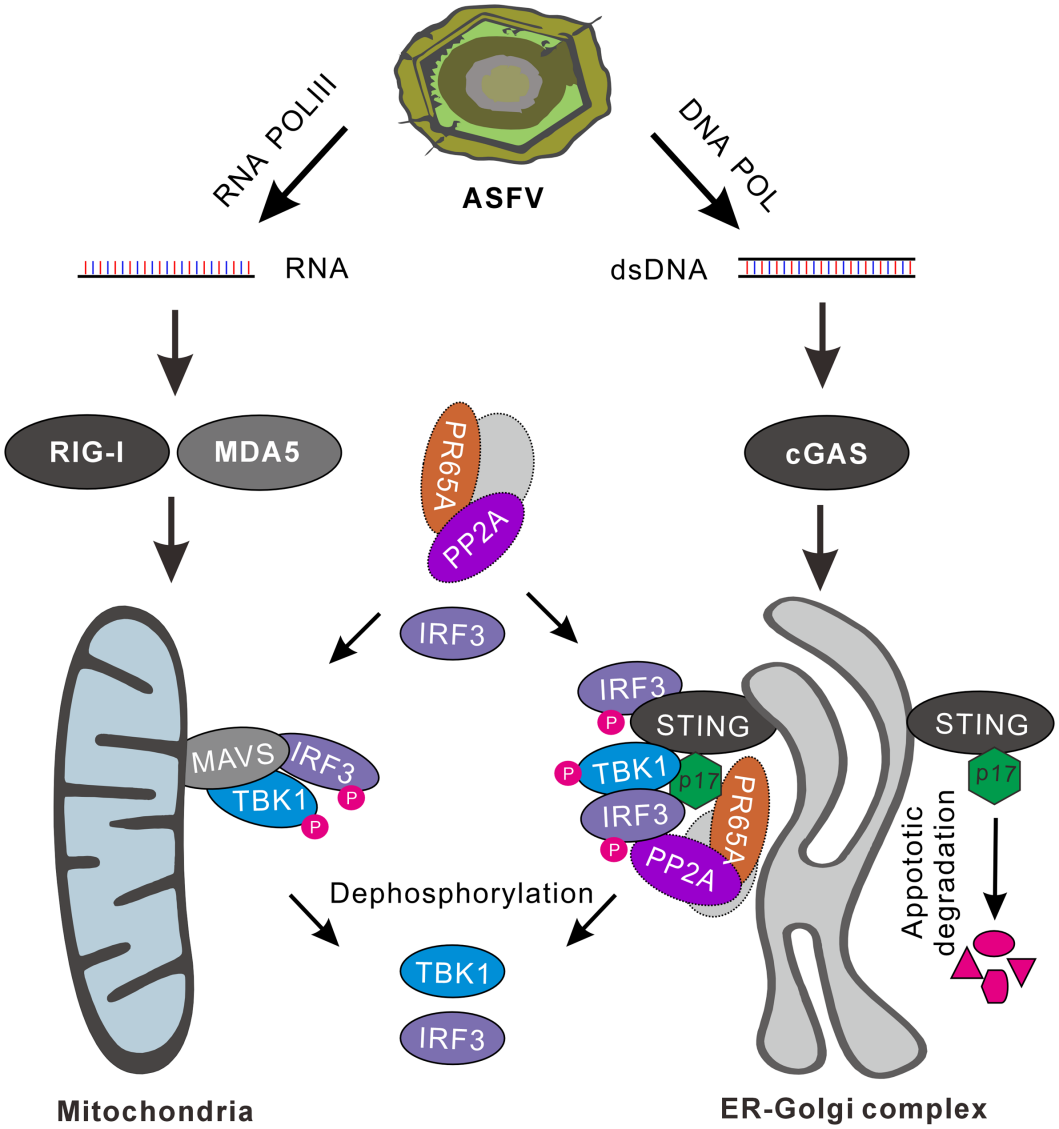
A proposed model for the inhibition of IFN-β signaling by ASFV p17. The AT-rich genome of ASFV can generate RNA and dsDNA through the activity of host RNA polymerase III, viral RNA transcriptase and DNA polymerase. Viral dsRNAs can activate both RLR-MAVS-NF-κB and RLR-MAVS-IRF3 pathways, whereas the dsDNA mainly targets cGAS-STING-IRF3 signaling. ASFV 17, an ER-Golgi localized membrane protein, can effect on IRF3 activation via several mechanisms. It can interact with STING and recruit PR65A, a subunit of cellular phosphatase PP2A, to dephosphorylate TBK1 and IRF3. It can recruit away IRF3 by interaction from MAVS-TBK-IRF3 signaling, and then down-regulate the level of p-IRF3 by PR65A. Additionally, p17 can facilitate STING degradation by induction of cellular apoptosis, leading to attenuation of STING-TBK1-IRF3 signaling transduction.

Overall, our studies in this report discover a preferential interaction of p17 with cGAS-STING-IRF3 signaling, but not the RIG-1-MAVS-NF-κB signaling, and that p17 executes function via several means, leading to down-regulation of IRF3 phosphorylation and the resulted attenuation of IFN-β. Figure 8 depicts a proposed model illustrating how p17 exerts its inhibitory function on the two signaling branches. The findings provide novel insights into the biological function of ASFV-encoded proteins and its intricate interplay with cellular interferon signaling.

## Data availability statement

The datasets presented in this study can be found in online repositories. The names of the repository/repositories and accession number(s) can be found in the article/Supplementary material.

## Ethics statement

Ethical approval was not required for the studies on humans in accordance with the local legislation and institutional requirements because only commercially available established cell lines were used. Ethical approval was not required for the studies on animals in accordance with the local legislation and institutional requirements because only commercially available established cell lines were used.

## Author contributions

JH: Conceptualization, Formal analysis, Funding acquisition, Supervision, Writing – review & editing. SW: Data curation, Formal analysis, Investigation, Methodology, Project administration, Validation, Writing – original draft. ZX: Investigation, Writing – original draft. PG: Investigation, Resources, Writing – original draft. YZ: Resources, Supervision, Writing – original draft. LZ: Resources, Supervision, Writing – original draft. XGe: Resources, Supervision, Writing – original draft. XGu: Resources, Writing – original draft. HY: Conceptualization, Formal analysis, Funding acquisition, Resources, Supervision, Writing – original draft.
